# Construction and Verification of a Predictive Nomogram for Overall Survival in Patients with Large Retroperitoneal Liposarcoma: A Population-Based Cohort Study

**DOI:** 10.3390/curroncol32080473

**Published:** 2025-08-21

**Authors:** Huan Deng, Zhenhua Lu, Yajie Wang, Lin Xiao, Yisheng Pan

**Affiliations:** 1 Department of Gastrointestinal Surgery, Peking University First Hospital, Beijing 100034, China; 2 Department of General Surgery, The First Medical Center, Chinese People’s Liberation Army General Hospital, Beijing 100853, China; 3 Key Laboratory of Carcinogenesis and Translational Research (Ministry of Education), Department of Gastrointestinal Surgery, Peking University Cancer Hospital & Institute, Beijing 100142, China

**Keywords:** retroperitoneal liposarcoma, large, surgery, nomogram, survival

## Abstract

Objective This study aimed to show the clinicopathological characteristics of large retroperitoneal liposarcoma (RLS) and to develop a customized nomogram model for patients with large RLS. Methods A total of 1735 patients diagnosed with RLS were selected from the public SEER database. Among them, 1113 patients with a maximum tumor diameter greater than 150 mm were included for further analysis. Nomogram models were developed based on Lasso and multivariate Cox regression analyses. A total of 166 patients that presented in the same period at our institution were used for external validations. Results A larger tumor size in RLS was associated with worse survival outcomes. Lasso and Cox regression analyses consistently identified age, TNM stage, occurrence pattern, histology, and surgery as important prognostic factors for OS. The constructed model demonstrated robust predictive performance, with better time-ROC (time-dependent receiver operating characteristic) for 1-year (83.1%), 3-year (83.8%), and 5-year (81.4%) survival in the training cohort. The concordance index (C-index) was approximately 0.80 in both the training and validation cohorts, reflecting excellent discriminatory ability of the model. Survival risk stratification analysis revealed significant differences in survival outcomes of large RLS (HR = 4.12 [3.31–5.12], *p* < 0.001, in the training cohort). Decision curve analysis (DCA) confirmed that the nomogram provided greater net benefits across a range of threshold probabilities. Conclusion This study identified important prognostic factors for survival in patients with large RLS and developed a reliable nomogram for predicting OS. The model’s strong predictive performance supports its use in personalized treatment strategies, improving prognosis assessment and clinical decision making for these patients.

## Simple Summary

This study presents the development and validation of a prognostic nomogram designed to predict overall survival in patients with large RLS. The nomogram was derived from a comprehensive analysis of clinical and pathological data from the SEER database, incorporating key prognostic factors such as age, TNM stage, tumor occurrence pattern, histology, and treatment methods. The model’s predictive performance was rigorously validated through various statistical analyses, demonstrating its reliability and utility in clinical practice for personalized treatment planning and enhanced patient outcomes.

## 1. Introduction

Retroperitoneal liposarcoma (RLS) is a rare malignancy that originates in the retroperitoneal space and represents the predominant form of retroperitoneal sarcoma [1]. It accounts for approximately 0.07% to 0.2% of all malignancies and 12% to 40% of all liposarcomas [2]. According to the WHO (World Health Organization) classification, the histology of RLS can be further categorized into four subtypes: well-differentiated liposarcoma (WDL), dedifferentiated liposarcoma (DDL), myxoid liposarcoma (MLS), and pleomorphic liposarcoma (PLS). Among these subtypes, WDL and DDL are the predominant subtypes, comprising about 37% to 56% of primary retroperitoneal liposarcomas [3,4]. The prognosis of RLS is influenced by its histological subtype, with poorly differentiated tumors generally linked to higher rates of local recurrence and distant metastasis [5].

Although resection remains the primary treatment for RLS, the tumor exhibits a higher tendency for relapse following surgical intervention [6]. According to the previous literature, the resection margin and histologic subtype are the most important prognostic predictors of RFS and OS for RLS [7]. A substantial body of research indicates that the combined resection of adjacent organs, such as renal and gastrointestinal tissues, can significantly improve local outcomes by reducing the risk of recurrence and enhancing the effectiveness of treatment [8,9,10]. However, the complex anatomical features of RLS pose significant challenges, often hindering the surgeon’s ability to achieve clear surgical margins, which is frequently associated with an unfavorable prognosis. Sometimes, complete capsule resection and radical surgical treatment cannot achieve a complete cure in RLS, which is a challenge for surgeons [11].

For larger RLS tumors, the extent of invasion and treatment challenges differ significantly from those with typical RLS. According to the eighth edition of the AJCC Cancer Staging Manual, tumors larger than 15 cm are classified as the T4 category [8]. Currently, there is no dedicated research addressing the survival prognosis of patients with T4 stage of RLS or the differences in clinical and pathological characteristics compared to those with smaller tumors. In clinical practice, we have observed that tumors with large volumes often invade complex abdominal structures, increasing surgical risks and difficulty [12,13]. The short-term and long-term prognoses of these patients differ significantly from those of patients with smaller tumors.

In our study, the term “large” was specifically defined as a tumor with a maximal diameter exceeding 150 mm (T4 stage) [13,14]. Compared to other types of RLS, a significant proportion of patients with large tumors have experienced rapid mortality, primarily due to the impacts of local recurrence or distant metastasis. The large tumor volume and its proximity to critical abdominal structures are key factors that distinguish large RLS in this disease, contributing to the increased complexity and difficulty in both diagnosis and treatment.

The aim of this study was to comprehensively analyze the clinicopathological features and prognostic outcomes of large RLS. We collected retrospective case data from public SEER databases and conducted multidimensional analyses and validations to provide valuable insights for the clinical treatment and prognosis assessment of patients with large RLS. Additionally, survival and prognostic models were assessed to further elucidate the clinical trajectory of this specific RLS. The findings contribute valuable evidence to support the development of personalized clinical management strategies for RLS.

## 2. Materials and Methods

### 2.1. Patient Selection

The data utilized in this study were derived from two primary sources. The first source was the public SEER database, accessed through the SEER*Stat software version 8.4.3 (USA) provided by the National Cancer Institute (https://seer.cancer.gov/data-software/, 25 June 2024). The second source was cases treated at the First Medical Center, CPLAGH, from 2000 to 2020. This study was approved by the Protection of Human Subjects Committee of the CPLAGH. The cases of second source served as external validations. The screening of patients with RLS in the SEER database is shown in Supplementary Figure S1. However, cases with distant organ metastasis were missing from the second source. Finally, a total of 166 patients with T4 stage of RLS were collected from the second source. We extracted key demographic information, clinicopathological characteristics, treatment methods, and vital survival status data from the SEER database. The primary endpoint of this study was OS.

### 2.2. Important Definitions

Overall survival (OS) was defined as the time from the surgery date to the time of the last follow-up or death. Primary RLS was defined as the initial diagnosis of an RLS tumor. Recurrent RLS was defined as an RLS tumor that relapsed at least once since the initial diagnosis. The definition of large RLS was specifically defined as a tumor with a maximal diameter exceeding 150 mm (T4 stage) [14]. WDL and MLS are histological low-grade tumors, and DDL and PLS are histological high-grade tumors [15]. The local and non-local occurrence patterns were defined according to the SEER manual (https://seer.cancer.gov/seerstat/variables/seer/lrd-stage/, accessed on 25 June 2024) and a previous study. Local occurrence was defined as the tumor’s origin site and main growth areas located within an abdominal compartment in this study [12,13]. The TNM stages were confirmed according to AJCC Retroperitoneal Soft Tissue Sarcoma Staging System (8th Edition, 2016) [16].

### 2.3. Statistical Analysis

Categorical data are presented as frequencies (percentages) and compared using either the chi-square test or Fisher’s exact test. The normality of continuous variables was assessed using the Shapiro–Wilk test. For variables that did not follow a normal distribution, data are reported as medians with interquartile ranges (IQRs, Q1–Q3). Differences between two independent groups were compared using the non-parametric Mann–Whitney U test.

The least absolute shrinkage and selection operator (Lasso) was used to explore optimal predictors for OS. Selection of the optimal tuning parameter (λ) in the Lasso regression model was performed using 10-fold cross-validation. The partial likelihood deviance is plotted against log(λ), and the optimal λ is determined at the point with the minimum mean cross-validated error. In the Lasso coefficient profiles, the vertical dashed line represents the optimal log(λ), where variables with non-zero coefficients are selected. Univariate Cox regression analysis was used to identify potential prognostic variables. Variables exhibiting multicollinearity, as indicated by a variance inflation factor (VIF) greater than 4, were excluded from further analysis. The remaining variables were then incorporated into the final multivariate Cox regression model. These models were constructed using the coxph function from the survival package in R. The prognostic variables identified from the training cohort were used to develop a nomogram for predicting the survival probability of patients at 1, 3, and 5 years. Each factor on the nomogram is linked to a specific point on the “Point” scale. The total score is obtained by summing the points for each variable. The probabilities of 1-, 3-, and 5-year OS are indicated by the intersection of the total point score with the corresponding bottom scales. Model calibration was conducted using the training set (70%), the internal validation set (30%), and an external cohort. The time-dependent ROC and C-index were used to validate the discrimination of the model [17].

Survival rates among different groups were compared using Kaplan–Meier survival curves. To assess the statistical significance of the differences between these groups, the log-rank test or Cox regression analysis was performed. Data analysis and visualization were conducted using R software (Version 4.2.2, Auckland, New Zealand, 16 March 2024), with a two-sided *p* value of <0.05 considered statistically significant.

## 3. Results

### 3.1. Clinicopathological Features and Survival Outcomes

Using the aforementioned inclusion criteria and procedures, a total of 1735 cases were extracted from the SEER database. Based on the previously defined classifications, 1113 cases were allocated to the large group, while 622 cases were assigned to the non-large group. The large group comprised 550 female and 613 male patients, whereas the non-large group included 251 female and 371 male patients. The median age for the large group was 63 years (IQR, 53–71), compared to 65 years (IQR, 55–73) for the non-large group. The median tumor size in the large group was 250 mm (IQR, 200–310), while in the non-large group, it was 100 mm (IQR, 68–130) (Table 1).

**Figure 1:**
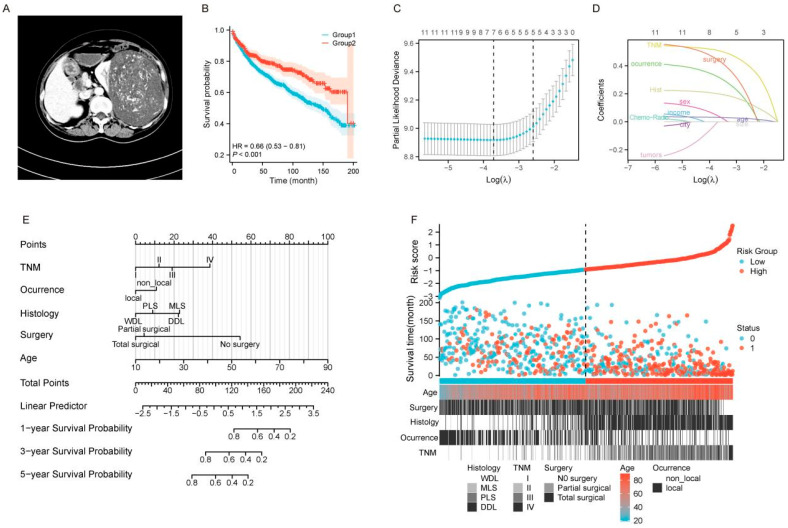
Prognostic analysis and nomogram development for patients with large RLS. (**A**) Preoperative imaging examinations of a case with large RLS. (**B**) Kaplan-Meier survival curves comparing OS between two groups (Group 1: Large group; Group 2: Non-group). (**C**) Selection of the optimal tuning parameter (λ) in the Lasso regression model using 10-fold cross-validation. The partial likelihood deviance is plotted against log(λ), and the optimal λ is determined at the point with the minimum mean cross-validated error. (**D**) Lasso coefficient profiles of the included variables. The vertical dashed line represents the optimal log(λ), where variables with non-zero coefficients are selected. (**E**) Nomogram for predicting 1-year, 3-year, and 5-year OS for patients with large RLS. (**F**) Risk score distribution and stratification of patients into low-risk and high-risk groups. The scatterplot demonstrates risk scores, survival times, and survival status (alive = 0, deceased = 1) along with key patient characteristics, including age, type of surgery, histology, TNM stage, and occurrence pattern (local vs. non-local). The dashed line separates the low-risk group from the high-risk group, as determined by the cutoff value. Abbreviations: OS, overall survival; TNM, tumor–node–metastasis; WDL, well-differentiated liposarcoma; PLS, pleomorphic liposarcoma; MLS, myxoid liposarcoma; DDL, dedifferentiated liposarcoma; RLS, retroperitoneal liposarcoma; Lasso, least absolute shrinkage and selection operator.

There were significant differences between the two groups in clinical and pathological characteristics, including AJCC TNM stage, occurrence pattern, pathological grade, occurrence sequence, and treatment outcomes (Table 1 and Figure 1A, *p* < 0.05). We found that patients with large tumors have worse survival outcomes (Figure 1B, *p* < 0.05, HR = 0.66 [95% CI: 0.53–0.81]). Subgroup analysis of overall survival in the large tumor group revealed that female patients tended to experience longer survival benefits (Supplementary Figure S2A). As age increased, the survival benefit for these patients gradually decreased (Supplementary Figure S2B). The number of tumors and the patients’ residing city did not show significant long-term survival differences in this analysis (Supplementary Figure S2C–D). However, these patients exhibited distinct characteristics in terms of occurrence patterns, chemotherapy/radiotherapy, pathological subtypes, TNM stages, and treatment outcomes (Supplementary Figure S2E–I, *p* < 0.001). Therefore, different clinical management strategies and personalized diagnosis and treatment should be given to patients with large RLS.

### 3.2. Survival Predictive Factor Screening and Nomogram Model Establishment

Building upon the previous study, we further investigated the prognostic factors associated with the large tumor. We randomly divided the cohorts into a training cohort and a validation cohort in a ratio of 7:3. A total of 779 patients were assigned to the training set, and 575 patients were allocated to the internal validation set (Supplementary Table S1). The divided cohorts were comparable in terms of demographic and clinical features (*p* > 0.05). To identify relevant clinical and pathological predictors of survival outcomes in the training cohort, we performed Lasso regression analysis. Variables with evident multicollinearity, such as T stage, N stage, M stage, and pathological grade, were excluded from the analysis. Following this, a total of 12 significant prognostic factors were selected and incorporated into the Lasso regression model for further evaluation (Figure 1C,D).

In the preliminary Lasso regression model, seven significant factors in the training cohort, i.e., age, sex, TNM stage, occurrence pattern, histology, tumor size, and surgery, were identified based on the value of λ_min_ (Figure 1C,D). To enhance the efficiency of prognostic model, we subsequently included the seven identified factors into the Cox regression model for further validation and refinement. The results indicated that sex and tumor size did not demonstrate significant prognostic value and were therefore excluded from the predictive model (Table 2, *p* < 0.05). Ultimately, the results of the Cox regression analysis for the five significant factors were consistent with those obtained from the Lasso regression (λ_1-se_, Figure 1C,D), demonstrating strong coefficients [18]. Therefore, age, TNM stage, occurrence pattern, histology, and surgery were subsequently incorporated into the final predictive model. Based on the results of the stepwise analysis, we constructed a reliable nomogram model to predict OS in patients with large RLS (Figure 1E).

### 3.3. Multidimensional Validation of the Predictive Model’s Performance

The survival prediction model, based on a large dataset, demonstrates robust prognostic value for patients with large tumors using only a few simple clinical indicators (Figure 1E). We visualized the risk scores of patients using a heatmap based on the survival risk assessment from the model. The heatmap clearly demonstrates that patients in the low-risk group generally have longer survival times, while those in the high-risk group exhibit shorter survival times, indicating the model’s strong risk stratification capability. Furthermore, the heatmap reflects the predictive value of the five prognostic factors in distinguishing survival outcomes between high-risk and low-risk groups (Figure 1F).

**Figure 2:**
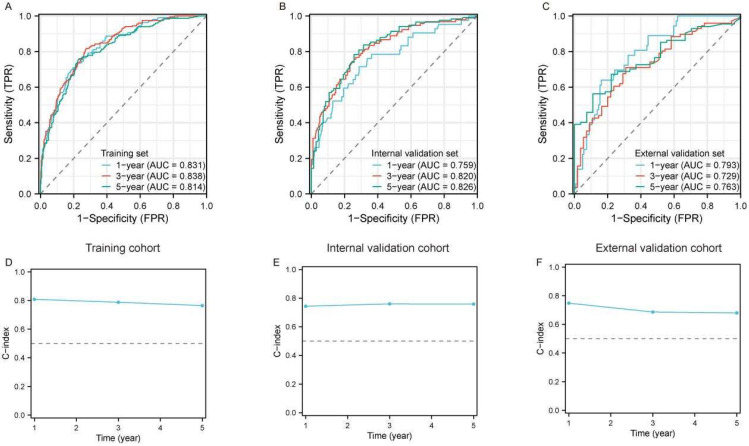
Evaluation of the predictive performance of the prognostic model for large RLS. (**A**–**C**) Time-dependent receiver operating characteristic (ROC) curves for 1-year, 3-year, and 5-year OS predictions in the training cohort (**A**), internal validation cohort (**B**), and external validation cohort (**C**). The area under the curve (AUC) values for each time point indicate the model’s discriminative ability. (**D**–**F**) Calibration of the model using the time-dependent concordance index (C-index) across the training cohort (**D**), internal validation cohort (**E**), and external validation cohort (**F**). The dashed horizontal line represents the reference level for model performance; Abbreviations: ROC, receiver operating characteristic; AUC, area under the curve; OS, overall survival; C-index, concordance index; RLS, retroperitoneal liposarcoma.

We further utilized time-dependent ROC curves to dynamically evaluate the discriminatory ability and performance of the constructed model, incorporating both survival outcomes and survival time. The results demonstrated that the model exhibited well predictive accuracy for 1-year (AUC = 83.1%), 3-year (AUC = 83.8%) and 5-year (AUC = 81.4%) survival in the training cohort (Figure 2A). Additionally, in both the internal (Figure 2B) and external validation sets (Figure 2C), the AUC values for 1-, 3-, and 5-year survival ranged from 72.9% to 83.8%, demonstrating robust predictive performance over time. Similarly, the time-dependent concordance index (C-index) was applied to validate the discrimination ability and efficiency of the model. We found that the C-index for 1-, 3-, and 5-year survival approached nearly 80% in the training cohort (Figure 2D) and both validation cohorts (Figure 2E,F). These results indicated favorable discrimination of the nomogram model.

**Figure 3:**
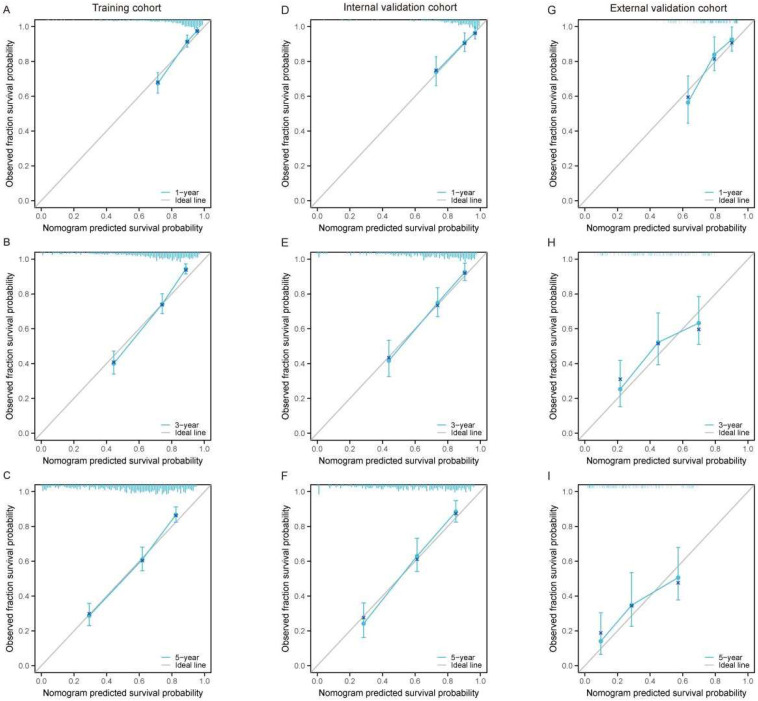
Calibration curves for the nomogram predicting 1-year, 3-year, and 5-year OS in large RLS across different cohorts. (**A**–**C**) Calibration curves for the training cohort at 1-year (**A**), 3-year (**B**), and 5-year (**C**) OS. (**D**–**F**) Calibration curves for the internal validation cohort at 1-year (**D**), 3-year (**E**), and 5-year (**F**) OS. (**G**–**I**) Calibration curves for the external validation cohort at 1-year (**G**), 3-year (**H**), and 5-year (**I**) OS. The x-axis represents the nomogram-predicted survival probability, while the y-axis represents the observed fraction survival probability. The solid diagonal line indicates the ideal prediction, where predicted probabilities perfectly align with observed outcomes. Blue lines show the model’s performance, with error bars representing the 95% confidence intervals. The proximity of the calibration curve to the diagonal line demonstrates the accuracy and reliability of the nomogram predictions. Abbreviations: OS, overall survival.

We further assessed the calibration ability of the nomogram by comparing the predicted survival probabilities with the actual survival probabilities using calibration plots. The model demonstrated overall good performance in the training set (Figure 3A–C), internal validation set (Figure 3D–F), and external validation set (Figure 3G–I), with the predicted survival probabilities for 1-year, 3-year, and 5-year survival closely aligning with the actual survival probabilities. This indicates that the nomogram exhibits good calibration. In the external validation set (Figure 3G–I), the overall trend remains reasonable, suggesting that the model has a certain degree of generalizability.

**Figure 4:**
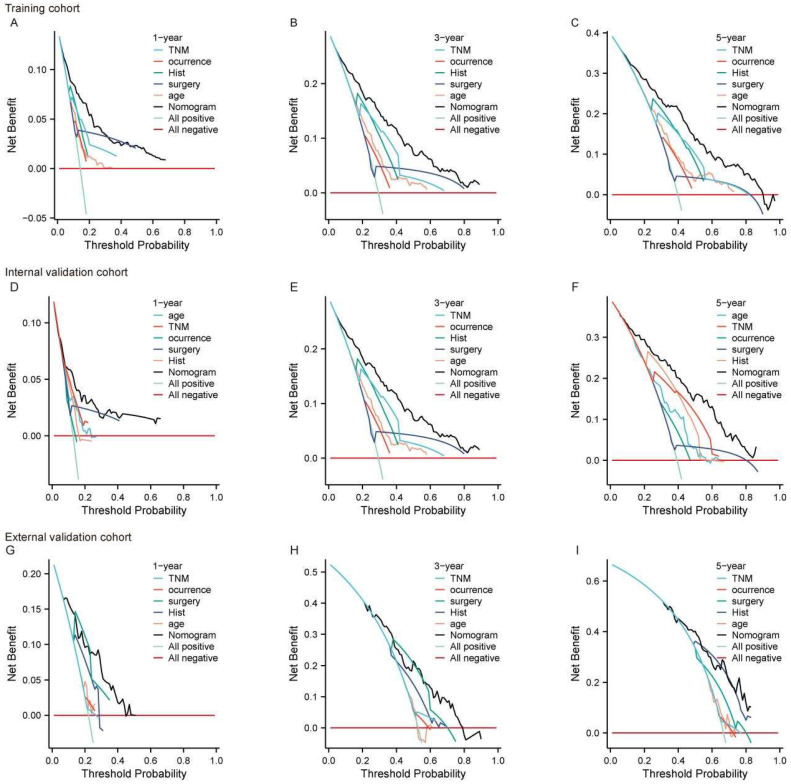
The DCA curve for the nomogram predicting 1-year, 3-year, and 5-year OS in large RLS across different cohorts. (**A**–**C**) DCA for the training cohort at 1-year (**A**), 3-year (**B**), and 5-year (**C**) OS. (**D**–**F**) DCA for the internal validation cohort at 1-year (**D**), 3-year (**E**), and 5-year (**F**) OS. (**G**–**I**) DCA for the external validation cohort at 1-year (**G**), 3-year (**H**), and 5-year (**I**) OS. The x-axis represents the threshold probability, while the y-axis represents the net benefit. The nomogram is compared to individual prognostic factors, including TNM stage, occurrence (local vs. non-local), histology (Hist), type of surgery, and age. The “All positive” line assumes all patients have the event, while the “All negative” line assumes none have the event. Abbreviations: OS, overall survival; TNM, tumor–node–metastasis; DCA, decision curve analysis; RLS, retroperitoneal liposarcoma.

We also utilized the DCA curve to assess the clinical utility of the predictive model. By calculating net benefit across various threshold probabilities, and balancing the trade-off between true positives and false positives, we evaluated the model’s practical value. The DCA curves were presented for different time points (1-year, 3-year, 5-year) and datasets. In the training set (Figure 4A–C), internal validation set (Figure 4D–F), and external validation set (Figure 4G–I), the black curve illustrates that the model’s overall predictive performance surpasses that of individual predictor variables. The nomogram consistently demonstrated superior net benefit at each time point and across all datasets. Notably, in the external validation set (Figure 4G–I), the nomogram maintained a higher net benefit, highlighting the model’s good generalizability and its ability to preserve predictive accuracy in independent datasets. Its consistent performance and high generalizability further substantiate its potential as a reliable tool for survival prediction and clinical decision making.

### 3.4. Survival Analysis Based on Risk Stratification

**Figure 5:**
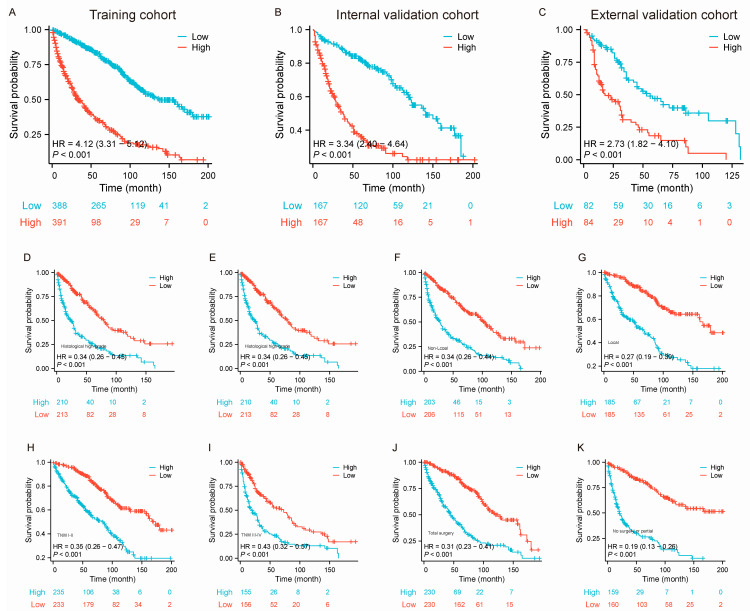
Kaplan–Meier survival curves stratified by risk groups and clinical characteristics for patients with large RLS in the training, internal validation, and external validation cohorts. (**A**–**C**) Kaplan–Meier survival curves comparing OS between low-risk and high-risk groups in the training cohort, internal validation cohort (**B**), and external validation cohort (**C**); (**D**–**K**) Kaplan–Meier survival curves comparing OS between low-risk and high-risk groups stratified by clinical characteristics, including histological subtypes (**D**–**E**), occurrences (non-local vs. local; (**F**–**G**), TNM stages (**H**–**I**), and types of surgery (total surgery, partial surgery, and no surgery; (**J**–**K**)). The number of patients at risk at each time point is shown below each curve. Abbreviations: OS, overall survival; TNM, tumor–node–metastasis; RLS, retroperitoneal liposarcoma.

The median OS of patients with large RLS in this study was approximately 89 months (interquartile range [IQR]: 79–97 months) (Supplementary Figure S3). We used the nomogram model to calculate the total points for each individual and further stratified them based on risk. To validate the effectiveness of the constructed nomogram model, patients with a large RLS were divided into high-risk and low-risk groups. We initially stratified all patients into high-risk and low-risk groups within the training set (Figure 5A, HR = 4.12 [3.31–5.12], *p* < 0.001), internal validation set (Figure 5B, HR = 3.34 [2.40–4.46], *p* < 0.001), and external validation set (Figure 5C, HR = 2.73 [1.82–4.10], *p* < 0.001). The results showed that patients in the high-risk group had significantly shorter survival times, indicating that the predictive model demonstrated strong risk stratification capability. Additionally, we conducted subgroup risk stratification analyses based on key clinicopathological characteristics (Figure 5D–K). The results showed that the model consistently exhibited strong predictive performance across all subgroups, underscoring its robust risk stratification capability. These findings highlight the model’s reliability in survival prediction and its potential to serve as a valuable tool for clinical prognosis assessment.

## 4. Discussion

To date, there are only a few case reports of large retroperitoneal liposarcoma available [19]. Only a few specialized surgeons have gained enough treatment experience from these patients. Although numerous studies have outlined the characteristics of RLS, this is the first large-scale, comprehensive study to provide a clinical analysis and develop a predictive nomogram model for large RLS. In the present study, we investigated the baseline and clinicopathological characteristics of large RLS and analyzed the prognostic factors of OS.

The 5-year overall survival of patients with ordinary RLS usually exceeds 50% in most reports. Noriyuki Masaki et al. reported that the 5-year overall survival rates of patients with WDL and the DDL/myxoid subtype were 100% and 67.4%, respectively [9]. Alessandro Gronchi et al. reported a series of 144 patients affected by retroperitoneal liposarcoma over a 10-year time span, and the 5-year OS was 61.2% [20]. In our study, we collected 1113 cases, making it a relatively large cohort, which allows for a more comprehensive set of findings. The 1-, 3-, 5-, and 10-year overall survival rates were about 86%, 71%, 60%, and 38%, respectively (Supplementary Figure S3). It can be observed that the survival rate for this group of patients after 5 years is relatively low. Compared to non-large RLS, we could see that non-large survival rates were obviously better than those with large tumors (Figure 1A). There are many different aspects involved in large RLS, such as the large tumor occupying almost the entire abdominal cavity, close to or even wrapping around large important blood vessels [13]. These complicated anatomic characteristics make the surgical procedure very difficult, thereby affecting the survival rate of this group of patients

These patients usually experience a complicated surgery, need combined resection, and have a long operation time and a high bleeding volume, which are factors related to serious postoperative complications [21]. In our previous study, we found that the median intraoperative bleeding volume reached 1500 mL for patients with giant RLS, and the median operation time was 280 min [13]. Many studies have shown that postoperative complications are closely associated with a poor prognosis [21,22,23,24]. In our previous study, we also found that postoperative complications significantly influenced OS. Specifically, complications graded II-IV (Clavien–Dindo) were associated with a shorter 5-year OS compared to grade I complications [13]. Actually, this is an indirect reflection of the complicated dilemma in treating large RLS, and serious complications mean that large tumors are difficult for surgeons.

In addition, some studies also illustrated that the RLS histologic subtypes have a core effect on prognosis [7,25,26]. Noriyuki Masaki et al. reported 40 recurrent retroperitoneal liposarcoma cases and 23 patients with initial WDL, and pathological progression (PP) to DDL was observed in the re-recurrent tumors [9]. This indicates that RLS has a relapse tendency and that low-grade tumors may progress to high-grade tumors after surgical treatment, and this phenomenon inevitably impacts the outcomes of patients with RLS. However, in patients with large RLS, the increase in tumor size somewhat diminishes the impact of histological subtypes on survival prognosis. In other words, pathological subtypes become less influential in predicting outcomes for these patients, which is a distinctive characteristic of patients with large RLS.

Our study investigated the clinicopathological characteristics of the large RLS and explored the significant prognostic factors that are correlated with OS. Lasso and multivariate Cox regression analysis revealed that age, TNM stage, occurrence pattern, histology, and surgery were significant prognostic factors for large RLS. Some results from the Cox regression analysis are consistent with those of previous studies [27]. In the preceding analysis, we have highlighted the significance of histology in the prognostic assessment of RLS, and explained how surgical complexity and postoperative complications influence prognosis [25]. Postoperative TNM staging emerges as a critical prognostic factor for patients with large RLS [8]. By incorporating key variables such as tumor size, lymph node metastasis, and distant metastasis, the TNM stage offers a more comprehensive evaluation of the essential characteristics and status of patients. For large RLS, the broader surgical resection often leads to the removal of a greater number of lymph nodes, highlighting the importance of conducting a detailed examination of these lymph nodes for accurate staging and prognosis.

In previous studies, numerous predictive models have been established, such as Sarculator [28]. This model incorporates well-standardized prognostic factors such as the FNCLCC grade and histology. However, in clinical practice within China, the FNCLCC staging is difficult to obtain, particularly during the preoperative evaluation phase. It requires the assessment of tumor histological grade and alterations in mitotic activity. In our study, we combined retrospective data from the SEER database with our single-center data; however, the information necessary for the FNCLCC grading was missing in this cohort. Consequently, we were unable to evaluate tumor staging and grading using the FNCLCC system. The TNM staging system is an internationally recognized and widely used standard for cancer staging, including for soft tissue sarcomas [29]. By employing the TNM staging system, our research findings align more closely with clinical practice and facilitate the application of these findings in clinical decision making. Thus, we developed this model based on a larger dataset while considering the practical clinical scenario, aiming to provide a reference for the treatment and prognostic evaluation of this patient population.

Adjuvant therapies are pivotal for the prognosis of soft tissue sarcoma (STS) patients, significantly enhancing cure rates and survival, particularly for high-risk individuals [30]. These treatments may downstage tumors or improve resection margins, potentially making the tumor resectable and thus enhancing both short- and long-term outcomes. However, the role of adjuvant therapies in RLS remains controversial, as preoperative radiotherapy and chemotherapy do not consistently outperform surgery alone according to some clinical studies [31,32]. For large RLS patients, systematic evidence supporting the benefits of adjuvant therapies is lacking. Acknowledging the impact of chemotherapy and radiotherapy, our study’s limitations due to extensive data missing prevented a comprehensive discussion of their specific efficacies, emphasizing the need for future comprehensive data collection to provide more precise prognostic insights.

### Study Limitations

Our study has several limitations. First, as a retrospective analysis primarily relying on medical records from our institution and the SEER database, it is inherently limited by the absence of prospective data. Second, critical therapeutic indicators, such as information on adjuvant therapies, were either missing or unknown for the majority of cases. Third, key pathological data, including tumor necrosis and mitotic count, which are essential for accurate grading, were unavailable for some cases in the external cohort, complicating the pathological assessments. Fourth, given the long study period, we did not address the potential impact of advancements in treatment techniques and other factors on prognosis. Finally, due to the limited number of cases in the external cohorts, 10-year validation data were not available, further limiting the robustness of our findings.

## 5. Conclusions

This study investigated the clinicopathological characteristics and survival outcomes of 1735 patients with large RLS. A prognostic nomogram for predicting OS was developed using Lasso and Cox regression analyses. The model’s predictive accuracy and robustness were validated through time-dependent ROC analysis and C-index, demonstrating excellent discriminatory ability across the training and validation cohorts. This rigorously validated nomogram offers a reliable tool for facilitating personalized treatment strategies and enhancing prognosis assessment in patients with large RLS, providing valuable insights for clinical decision making.

## Figures and Tables

**Table 1:** The clinical and pathological characteristics of two groups in RLS.

Characteristics	Large RLS Group	Non-Large RLS Group	*p* Value
	1113	622	
Age, median (IQR)	63 (53, 71)	65 (55, 73)	<0.001
Tumor size, median (IQR)	250 (200, 310)	100 (68, 130)	<0.001
Sex, *n* (%)			0.065
Female	500 (44.9%)	251 (40.4%)	
Male	613 (55.1%)	371 (59.6%)	
Income, *n* (%)			0.816
High	505 (45.4%)	292 (46.9%)	
Middle	409 (36.7%)	223 (35.9%)	
Low	199 (17.9%)	107 (17.2%)	
City, *n* (%)			0.343
Metropolitan	997 (89.6%)	566 (91%)	
Nonmetropolitan	116 (10.4%)	56 (9%)	
AJCC T stage, *n* (%)			<0.001
T4	1113 (100%)	0 (0%)	
T3	0 (0%)	288 (46.3%)	
T1	0 (0%)	110 (17.7%)	
T2	0 (0%)	224 (36%)	
AJCC N stage, *n* (%)			0.026
N0	1094 (98.3%)	601 (96.6%)	
N1	19 (1.7%)	21 (3.4%)	
AJCC M stage, *n* (%)			0.454
M0	1048 (94.2%)	591 (95%)	
M1	65 (5.8%)	31 (5%)	
AJCC TNM stage, *n* (%)			0.012
III	374 (33.6%)	180 (28.9%)	
II	68 (6.1%)	62 (10%)	
I	596 (53.5%)	341 (54.8%)	
IV	75 (6.7%)	39 (6.3%)	
Radiotherapy, *n* (%)			0.101
No/Unknown	845 (75.9%)	450 (72.3%)	
Yes	268 (24.1%)	172 (27.7%)	
Chemotherapy, *n* (%)			0.807
No/Unknown	962 (86.4%)	535 (86%)	
Yes	151 (13.6%)	87 (14%)	
Occurrence pattern, *n* (%)			0.014
Non-local	596 (53.5%)	295 (47.4%)	
Local	517 (46.5%)	327 (52.6%)	
Pathological grade, *n* (%)			0.009
Undifferentiated	205 (18.4%)	110 (17.7%)	
Moderately differentiated	75 (6.7%)	37 (5.9%)	
Poorly differentiated	185 (16.6%)	87 (14%)	
Well differentiated	491 (44.1%)	259 (41.6%)	
Unknown	157 (14.1%)	129 (20.7%)	
Occurrence sequence, *n* (%)			<0.001
Recurrence	205 (18.4%)	168 (27%)	
Primary	908 (81.6%)	454 (73%)	
Tumors, *n* (%)			0.133
Single	1077 (96.8%)	593 (95.3%)	
Multifocal	36 (3.2%)	29 (4.7%)	
Histology, *n* (%)			0.916
DDL	586 (52.7%)	338 (54.3%)	
WDL	445 (40%)	238 (38.3%)	
MLS	57 (5.1%)	32 (5.1%)	
PLS	25 (2.2%)	14 (2.3%)	
Surgery, *n* (%)			<0.001
Total surgical	660 (59.3%)	267 (42.9%)	
partial surgical	385 (34.6%)	291 (46.8%)	
No surgery	68 (6.1%)	64 (10.3%)	

Abbreviations: TNM, tumor–node–metastasis; WDL, well-differentiated liposarcoma; PLS, pleomorphic liposarcoma; MLS, myxoid liposarcoma; DDL, dedifferentiated liposarcoma; RLS, retroperitoneal liposarcoma.

**Table 2:** The univariate and multivariate Cox regression analyses on significant factors in Lasso regression.

Characteristics	Total (*N*)	Univariate Analysis		Multivariate Cox Analysis	
		Hazard Ratio (95% CI)	*p* Value	Hazard Ratio (95% CI)	*p* Value
Age	779	1.041 (1.032–1.050)	<0.001	1.035 (1.026–1.045)	<0.001
Sex	779				
Male	417	Reference		Reference	
Female	362	0.736 (0.600–0.902)	0.003	0.852 (0.691–1.051)	0.134
TNM	779				
I	422	Reference		Reference	
II	46	1.828 (1.167–2.862)	0.008	1.384 (0.852–2.249)	0.189
III	259	2.568 (2.050–3.218)	<0.001	1.682 (1.233–2.295)	0.001
IV	52	5.536 (3.966–7.728)	<0.001	2.847 (1.929–4.204)	<0.001
Occurrence pattern	779				
Non-local	409	Reference		Reference	
Local	370	0.533 (0.433–0.656)	<0.001	0.729 (0.584–0.911)	0.006
Tumors	779				
Single	756	Reference		Reference	
Multifocal	23	0.600 (0.320–1.126)	0.112	0.791 (0.419–1.494)	0.470
Histology	779				
WDL	315	Reference		Reference	
MLS	41	2.187 (1.432–3.341)	<0.001	1.942 (1.259–2.997)	0.003
PLS	20	1.765 (0.952–3.271)	0.071	1.277 (0.659–2.475)	0.468
DDL	403	2.940 (2.344–3.687)	<0.001	1.858 (1.360–2.537)	<0.001
Surgery	779				
No surgery	50	Reference		Reference	
Partial surgical	269	0.176 (0.123–0.253)	<0.001	0.238 (0.163–0.348)	<0.001
Total surgical	460	0.200 (0.142–0.280)	<0.001	0.210 (0.145–0.303)	<0.001

Abbreviations: TNM, tumor–node–metastasis; WDL, well-differentiated liposarcoma; PLS, pleomorphic liposarcoma; MLS, myxoid liposarcoma; DDL, dedifferentiated liposarcoma; CI, confidence interval.

## Data Availability

Data analyzed in the study are available upon request pending application and authority approval. Requests to access the datasets should be directed to Yisheng Pan, bdyypanyisheng@163.com.

## Supplementary Materials

The following supporting information can be downloaded at: https://www.mdpi.com/article/10.3390/curroncol32080473/s1. Table S1: Comparison of clinical pathological features between validation cohort and training cohort; Figure S1: The flowchart for screening RLS patients from the SEER Database; Figure S2: Kaplan-Meier survival curves analyzing the impact of demographic and clinical characteristics on OS in patients with large RLS; Figure S3: Kaplan-Meier survival curves analyzing the OS in patients with large RLS.
