# Supplementary material for: Construction and Verification of a Predictive Nomogram for Overall Survival in Patients with Large Retroperitoneal Liposarcoma: A Population-Based Cohort Study
Source: Curr Oncol. 2025 Aug 21;32(8):473. doi: 10.3390/curroncol32080473 (PMC12385397; doi:10.3390/curroncol32080473)
Supplement: Supplementary file 1 [file curroncol-32-00473-s001.zip › curroncol-3737497-supplementary.pdf]

Supplementary materials

Table S1. Comparison of clinical pathological features between validation cohort and training cohort.

Characteristics	Training cohort	Validation cohort	P value
	779	334	
Age, median (IQR)	62 (53, 71)	63 (54, 71)	0.825
Size, median (IQR, mm)	250 (200, 310)	240 (190, 300)	0.076
Sex, n (%)			0.113
female	362 (46.5%)	138 (41.3%)	
male	417 (53.5%)	196 (58.7%)	
Income, n (%)			0.083
high	350 (44.9%)	155 (46.4%)	
middle	277 (35.6%)	132 (39.5%)	
low	152 (19.5%)	47 (14.1%)	
City, n (%)			0.095
metropolitan	690 (88.6%)	307 (91.9%)	
nonmetropolitan	89 (11.4%)	27 (8.1%)	
TNM, n (%)			0.923
III	259 (33.2%)	115 (34.4%)	
II	46 (5.9%)	22 (6.6%)	
I	422 (54.2%)	174 (52.1%)	
IV	52 (6.7%)	23 (6.9%)	
Chemo/Radio, n (%)			0.235
no/unknown	515 (66.1%)	233 (69.8%)	
yes	264 (33.9%)	101 (30.2%)	
Ocurrence pattern, n (%)			0.285
non_local	409 (52.5%)	187 (56%)	
local	370 (47.5%)	147 (44%)	

Characteristics	Training cohort	Validation cohort	P value
Ocurrence sequence, n (%)			0.935
recurrence	143 (18.4%)	62 (18.6%)	
primary	636 (81.6%)	272 (81.4%)	
Tumors, n (%)			0.417
single	756 (97%)	321 (96.1%)	
mutifocal	23 (3%)	13 (3.9%)	
Histology, n (%)			0.606
DDL	403 (51.7%)	183 (54.8%)	
WDL	315 (40.4%)	130 (38.9%)	
MLS	41 (5.3%)	16 (4.8%)	
PLS	20 (2.6%)	5 (1.5%)	
Surgery, n (%)			0.804
Total surgical	460 (59.1%)	200 (59.9%)	
partial surgical	269 (34.5%)	116 (34.7%)	
N0 surgery	50 (6.4%)	18 (5.4%)	

Abbreviations: TNM, tumor-node-metastasis; WDL, well-differentiated liposarcoma; PLS, pleomorphic liposarcoma; MLS, myxoid liposarcoma; DDL, dedifferentiated liposarcoma.

Supplementary figures

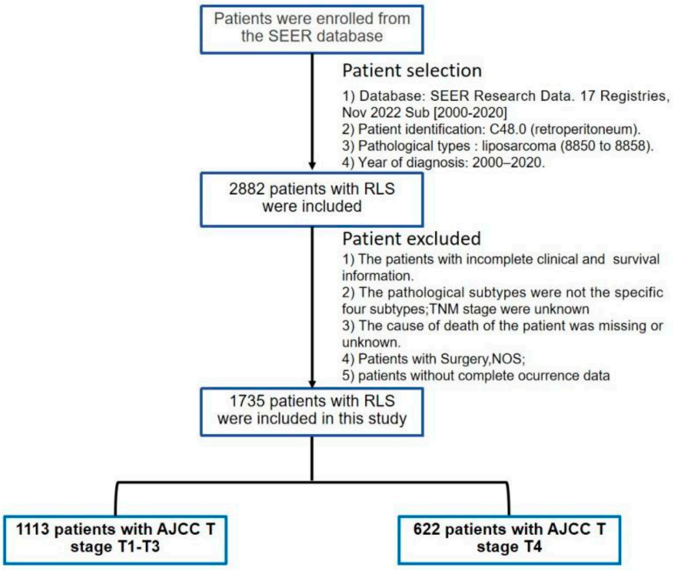

Figure S1. The flowchart for screening RLS patients from the SEER Database. Abbreviations: SEER, Surveillance, Epidemiology, and End Results; RLS, retroperitoneal liposarcoma.

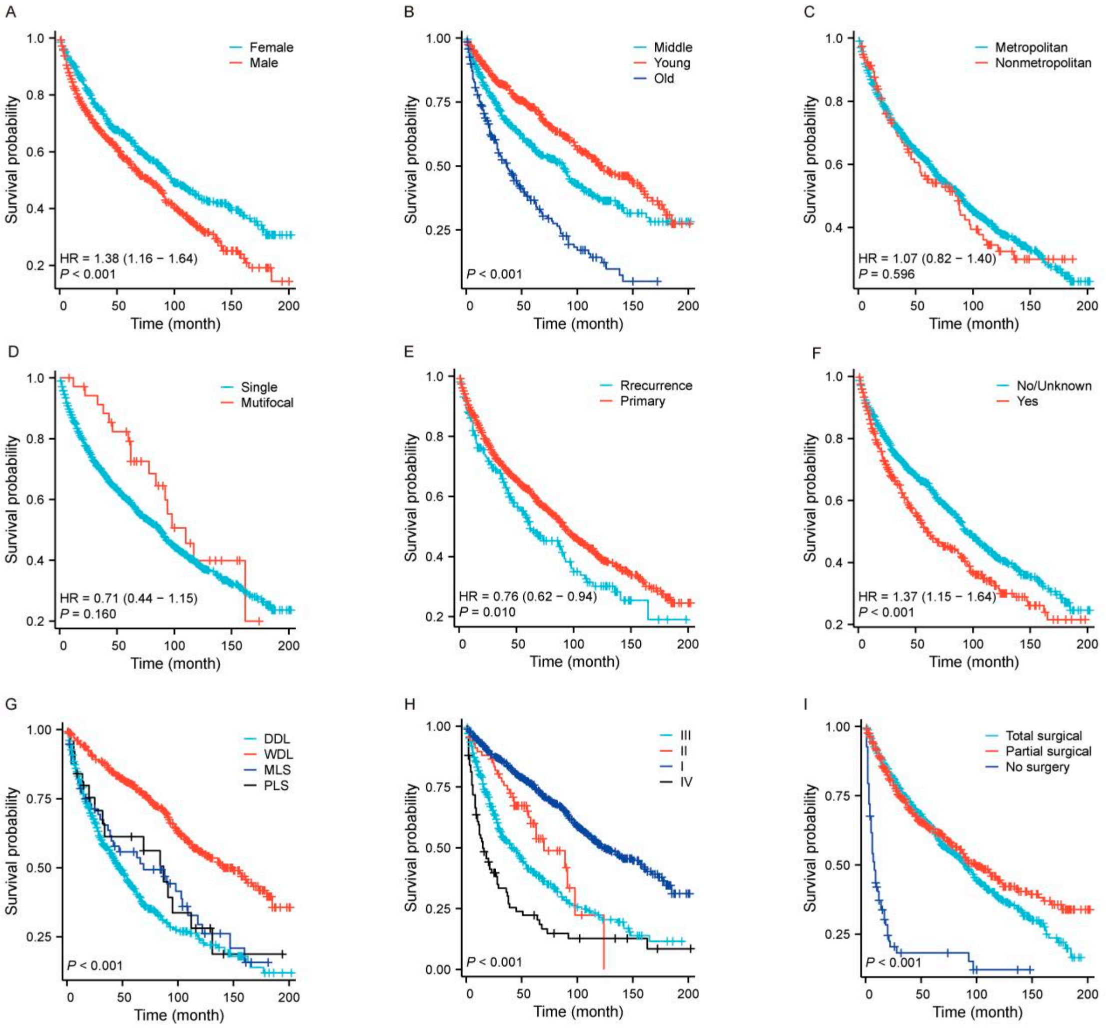

Figure S2. Kaplan-Meier survival curves analyzing the impact of demographic and clinical characteristics on OS in patients with large RLS. (A) Survival curves stratified by gender (female vs. male); (B) Survival curves stratified by age group (young, middle, old); (C) Survival curves stratified by residence type (metropolitan vs. non-metropolitan); (D) Survival curves stratified by tumor numbers (single vs. multifocal); (E) Survival curves stratified by tumor occurrence pattern (primary vs. recurrence. (F) Survival curves stratified by chemotherapy status (yes vs. no/unknown). (G) Survival curves stratified by histological subtypes (WDL, DDL, MLS, PLS); (H) Survival curves stratified by TNM stages (I-IV); (I) Survival curves stratified by type of surgery (total surgical, partial surgical, no surgery). Abbreviations: OS, overall survival; WDL, well-differentiated liposarcoma; DDL, dedifferentiated liposarcoma; MLS, myxoid liposarcoma; PLS, pleomorphic liposarcoma; TNM, tumor-node-metastasis; RLS, retroperitoneal liposarcoma.

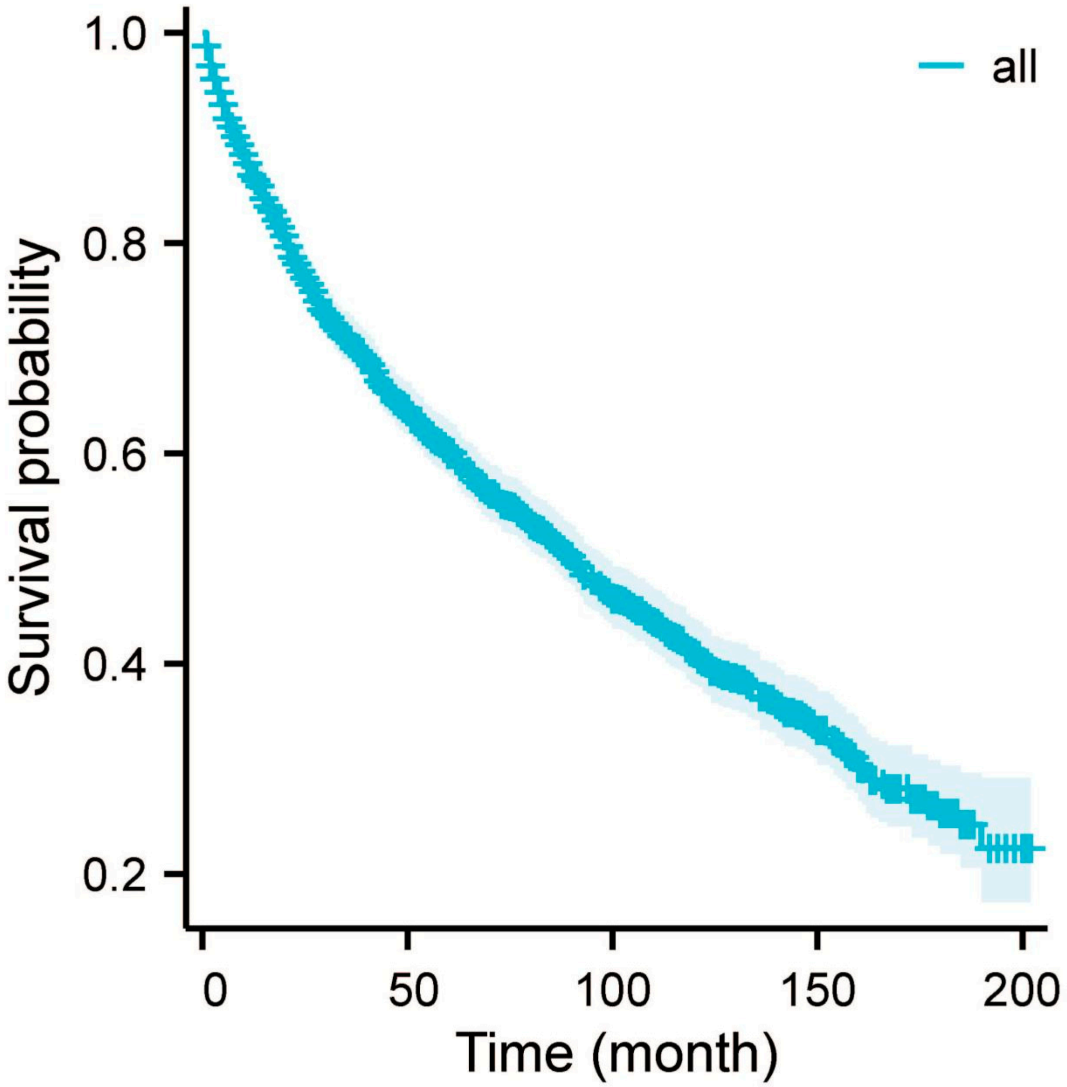

Figure S3. Kaplan-Meier survival curves analyzing the OS in patients with large RLS.

Abbreviations: OS, overall survival; RLS, retroperitoneal liposarcoma.
